# A custom-made mouthguard reduces head acceleration during soccer heading and prevents acute electrophysiological and cognitive changes in amateur male players

**DOI:** 10.1016/j.ebiom.2025.105674

**Published:** 2025-04-02

**Authors:** Claire Pitteu, Philippine Lepère, Philippe Poisson, Etienne Guillaud, Emilie Doat, Bertrand Glize, Patrick Dehail, Hélène Cassoudesalle

**Affiliations:** a Physical and Rehabilitation Medicine Department, University Hospital of Bordeaux, Place Amelie Raba Leon, 33000, Bordeaux, France; b University of Bordeaux, INSERM, BPH, U1219, ACTIVE Team, 146 Rue Léo Saignat, F-33000, Bordeaux, France; c Oral Medicine Department, University Hospital of Bordeaux, Avenue du Haut Lévêque, 33604, Pessac, France; d University Bordeaux, CNRS, INCIA, UMR 5287, F-33000, Bordeaux, France

**Keywords:** Soccer, Traumatic brain injuries, Mouthguard, Biomechanics, Transcranial magnetic stimulation, Cognition

## Abstract

**Background:**

Repetitive head impacts related to soccer heading might lead to long-term risk of neurodegenerative diseases. Helping to stabilise the head-neck-torso complex could be a preventive strategy. This study aims to investigate head acceleration and heading-related acute electrophysiological and cognitive changes in soccer players, with a custom-made mouthguard (CM-MG) and without.

**Methods:**

In this crossover study, 18 amateur male soccer players (age 22 ± 3 y) performed ten consecutive headers from machine-projected balls, with and without a CM-MG. Head accelerations during heading were recorded. Peak force of neck muscles were assessed. Before and immediately after heading, cognitive function was assessed, and the cortical silent period (cSP) was measured from the motor-evoked potentials recorded using Transcranial Magnetic Stimulation.

**Findings:**

A decrease of 6·40 g (95% CI [−11·74; 39·09]) of mean peak linear head acceleration was found with the CM-MG (26·43 g) compared to without (34·15 g, p = 0·01). The peak force of the head flexor muscles was higher with the CM-MG (172 N) than without (146·7 N), with a mean difference of 19·33 N (95% CI [13·39; 25·27]) (p < 0·001). The difference in mean cSP between pre- and post-heading decreased statistically significantly with the CM-MG (−9·17 ms) compared to without (20·03 ms; p = 0·0016), with a median difference of −22·87 ms (95% CI [−45·85; −4·02]). There was also a decrease in the changes to memory performance with the CM-MG versus without.

**Interpretation:**

A CM-MG may have the potential to protect the brain during soccer heading. More studies are still needed to confirm its benefits in all playing conditions on the field.

**Funding:**

This research received two grants from 10.13039/501100020976LabEx BRAIN- Bordeaux University and France Traumatisme Cranien (public sector).

**Research in context**

**Evidence before this study**

The sources were searched in MEDLINE, EMBASE, CENTRAL, CINAHL, OPENGREY, OPENSIGLE, PEDRO, WEB OF SCIENCE, CLINICAL TRIALS, from the beginning of the databases to June 2020 for designing this protocol, and until July 2024 for writing this paper.

The search terms used were (soccer OR sport OR football) AND (heading OR “Traumatic Brain Injuries” OR concussion) AND ((acceleration OR biomechanics OR mouthguard OR neck OR “cervical muscle”) OR (“transcranial magnetic stimulation” OR EMG OR “corticomotor inhibition”) OR (cognition)).

Most studies had methodological limitations and therefore possible biases, such as a simple estimation of exposure to head-ball impacts (self-assessment by the players themselves), a lack of consideration of associated concussions, different target populations in terms of level of play, age, sex, and which varied according to the studies.

**Added value of this study**

While a previous experimental study found a transient increase of corticomotor inhibition and decrease in memory performance in soccer players immediately after twenty consecutive headers, our findings also showed such acute changes in corticomotor inhibition and cognitive data immediately after performing only ten consecutive soccer headers, which is closer to the average number of headers performed per player in a match.

Moreover, these changes were not found when the soccer players wore a custom-made mouthguard (CM-MG) specially designed to promote mandibular wedging (clenched jaw position). Evidence of a significant decrease in peak linear head acceleration during the heading protocol was found when wearing the CM-MG, and an increase in peak force of the head flexor muscles was associated with wearing the CM-MG. Thus, jaw clenching and more balanced force ratio between the head flexors and extensors with this equipment could partly explain the changes in acceleration of the head during soccer heading.

**Implications of all the available evidence**

Reducing head acceleration during sports-related head impacts might be crucial for preventing degenerative brain injuries. It remains unknown whether this effect varies with the type of head acceleration (e.g., linear and/or rotational), or when considering other numbers of impacts over longer periods of time. Future studies exploring the effects of soccer heading on the brain when wearing and not wearing CM-MGs in all playing conditions on the field are needed.

## Introduction

Soccer is known as the most popular sport in the world, with over 265 million players in more than 200 countries.^1^ ‘Heading’ the ball is a special feature of soccer characterised by an intentional head impact to control the ball during game, learnt by the players from an early age and repeated throughout their career. Ball-head impacts occur an average of 6–12 times per player per match.^2^^,^^3^ However, studies based on self-reported questionnaires show highly variable average frequencies of heading in soccer: from ten headers per player per match among professionals,^4^ to 800 headers per player per season,^5^ to 2000 headers per player over a career,^6^ and up to 44 headers per player over two weeks of combined match and training practice among amateurs.^7^ Exposure also varies depending on the player's position.^8^ Previously, we identified through video analysis during a semi-professional soccer season an average of 3·4 (±2·5) headers per hour of play per player, ranging from 0 to 9 depending on the individual, with two positions being more exposed: forwards and central defenders.^9^

A review of studies exploring the biomechanics of head and neck movements in American football and soccer highlighted that the primary factor associated with the risk of brain injuries (contusion, concussion, intracranial haematoma, axonal injuries) following a head impact is the head acceleration during the impact, rather than the impact force itself.^10^ According to the 6th International Conference on Concussion in Sport, “Sport-related concussion is a traumatic brain injury caused by a direct blow to the head, neck or body resulting in an impulsive force being transmitted to the brain that occurs in sports and exercise-related activities. This initiates a neurotransmitter and metabolic cascade, with possible axonal injury, blood flow change, and inflammation affecting the brain. Symptoms and signs may present immediately or evolve over minutes or hours, and commonly resolve within days, but may be prolonged.”^11^ The risk of concussion is linked to the maximum acceleration of the head, with a 50% risk of concussion at speeds of 750 m/s^2^ (or 76·5 g).^12^^,^^13^ Rotational forces, expressed as angular velocity in radians per second squared (rad/s^2^), can also result in concussions beyond a threshold estimated to be between 3500 and 5000 rad/s^2^.^13^ Biomechanical analyses have not convincingly shown that intentional soccer heading results in a significant risk of concussion, with an average linear acceleration peak of 14–23 g and an impact duration of 14–33 ms, and average angular acceleration peaks of 1400–2410 rad/s^2^.^13^ Nevertheless, the risk of concussion remains possible in the event of accidental impacts with the ball, or when the ball is not suited to the size of the player's head, or when the heading technique is poorly executed.^8^^,^^14^

Even though intentional soccer heading would not result in symptomatic concussion, the accelerations generated by the transfer of force on impact may cause “sub-concussive” injuries, defined as events occurring on head impact with insufficient force to produce clinical symptoms characteristic of concussion.^3^^,^^15^ The long-term effects of chronic exposure to repetitive concussive and sub-concussive head impacts are increasingly reported, with a risk of neurodegenerative disorders, including Alzheimer's disease or Chronic Traumatic Encephalopathy.^16^ Neuroimaging studies have revealed brain alterations in athletes with concussion, but also in athletes exposed to sub-concussive head impacts, such as American football players, even without a clinical diagnosis of concussion, and some were correlated with repetitive head acceleration event exposure.^17^, ^18^, ^19^, ^20^ Regarding studies focussing on soccer heading, alterations of the white matter microarchitecture were described in soccer players without a history of concussion, either compared to unexposed athletes or associated with the estimated number of headers previously performed.^21^^,^^22^ Marked cerebrovascular reactivity changes were observed in female asymptomatic soccer players associated to repetitive head acceleration events.^23^^,^^24^ Changes in resting-state functional brain connectivity associated with the number of headers over one season of men's semi-professional soccer were also reported.^25^ Moreover, epidemiological studies showed an increased mortality by neurodegenerative disease in professional soccer players.^26^, ^27^, ^28^

Besides, acute and transient electrophysiological changes post soccer heading were found previously using Transcranial Magnetic Stimulation (TMS).^29^ In particular, using TMS motor evoked potentials (MEPs) and cortical silent period (cSP) measurement, an increase in corticomotor inhibition was found in soccer players immediately after a series of twenty consecutive headers. TMS has already demonstrated utility in quantifying electrophysiological changes in concussion, and corticomotor inhibition seems to be the most reliable and reproducible marker of concussion, expressed by a longer cSP after a MEP.^30^, ^31^, ^32^ After a clinical diagnosis of concussion, longer cSP persisted beyond 24 h and sometimes up to two months depending on the study. In the study by Di Virgilio and colleagues, longer cSP was also found in the hours following the 20 headers performed by soccer players, but without any associated symptoms, and did not persist beyond 24 h. Only a transient decrease in memory performance was also found for less than 24 h post heading.^29^ So, this study showed acute electrophysiological changes, strengthening the evidence for direct, despite subclinical, consequences of soccer headers on brain function.

Thus, given the growing concern, ways are being sought to minimise head acceleration during soccer heading to prevent injuries: a review of the literature found that head-neck-torso alignment and balanced head flexor and extensor strength decrease linear and angular acceleration during purposeful heading.^10^ Indeed, biomechanical studies in soccer found that just prior to impact, the neck muscles act to stabilise the head to dissipate the effects of contact with the ball and that more than flexors or extensors strength, a balanced ratio between head flexors and extensors could decrease head acceleration, by damping flexion/extension oscillations experienced by the head during soccer heading.^10^^,^^33^^,^^34^ The neck musculature plays an important part in the neck-torso complex stabilisation.^33^, ^34^, ^35^ According to Euler's law of motion, force equals mass times acceleration, based on formula F (N) = m (mass) x A (acceleration). Stabilising head and neck, the effective mass of head-neck complex will increase and therefore, for a constant force, acceleration will be reduced.^35^ Furthermore, laboratory studies on skull models and with a pendulum impact testing device have showed that wearing a mouthguard was associated with reduced linear acceleration of the head during impacts.^36^ In rugby players, wearing a mouthguard was associated with an increase in sternocleidomastoid muscle activity and a significant decrease in head linear accelerations.^37^ The hypothesis suggested is that by applying pressure to the mouthguard with the teeth, the player contracts the neck muscles, thereby stabilising the skull during head impact and reducing the g-forces at which the brain may be exposed.^37^ In soccer, a study also found a significant decrease in linear accelerations during heading in 11 soccer players when clenching while wearing a mouthguard, compared to clenching without mouthguard and even more compared to no clenching and no mouthguard.^38^ Focussing on mouthguards in soccer is all the more relevant as soccer is considered to be one of the main risk sports with ball in terms of maxillofacial injuries and would present a high risk similar to that of rugby.^39^ However, their benefit in preventing the brain effects of soccer heading has not been demonstrated.

**Fig. 1:**
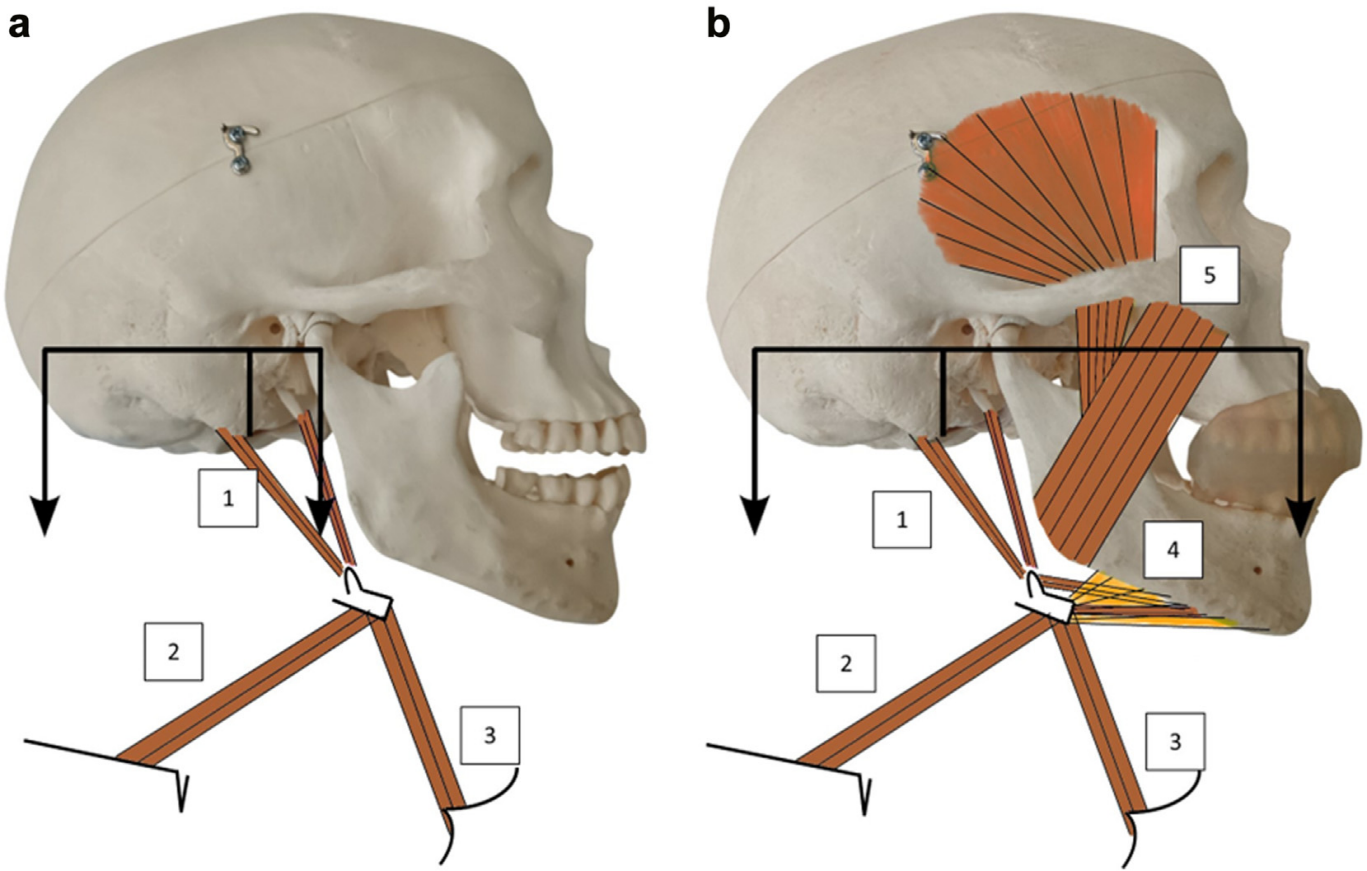
**Hypothetical mechanisms by which the custom-made mouthguard (CM-MG) reduces head acceleration**: **craniocervical flexion induced by the contraction of the Supra-Hyoid (SH) and Infra-Hyoid (IH) muscle groups**. a)Without CM-MG: (1) represents cranial insertion of SH muscles (stylohyoid and digastric), (2) and (3) represent the insertion of the IH muscles at the scapula and sternum, respectively. b) With CM-MG: The mandibular insertion of the SH muscles (digastric, mylohyoid, geniohyoid) (4) increases the length of the lever arm supported at the level of the occipital condyles (black), when the mandible is fixed via the CM-MG by the contraction of the powerful masticatory muscles (masseters and temporal) (5).

In this context, while unimaxillary mouthguard models are the most widespread, we focused on a bi-maxillary custom-made mouthguard (CM-MG), i.e. a one-piece device that covers both dental arches with the exception of the mandibular incisors and canines. This bi-maxillary CM-MG has the specificity of being able to maintain the mandible in a rest position, while promoting oral ventilation with clenched jaws during a sporting activity. The mandibular rest position is the natural, relaxed position of the lower jaw (mandible) when a person is at rest, not chewing, speaking, or clenching their teeth, and it represents the state of balance between the muscles that elevate and depress the mandible.^40^ In this position, the teeth of the upper and lower jaws do not touch. This position makes it possible to create an airway space in the CM-MG and consequently maintains mouth-breathing when the jaws are clenched, thus allowing mandibular support while providing supplementary oral ventilation for high-intensity efforts.^41^ Indeed, in some high-risk concussion sports, expired gases are higher than 30–40 L/min, so that nasal ventilation becomes insufficient and oral ventilation becomes necessary.^42^^,^^43^ We have already demonstrated the effect of mandibular wedging obtained with this CM-MG model in the prevention of oral and mandibular traumatology in high-risk sports, limiting the risk of “mandibular projectile” impacting the base of the skull, as well as its repercussions on motor control.^44^^,^^45^ Now, we hypothesised that mandibular wedging with CM-MG clenching, activating the anterior neck muscles through contraction of the masticatory muscles, might also contribute to better stabilisation of the skull and thus reduce head accelerations during head impacts, particularly during soccer heading. More precisely, the hypothetical mechanisms by which this CM-MG reduces head acceleration are as follows: 1) There is an increase in activation of the anterior neck muscles when the subject clenches his jaws on the CM-MG by the powerful contraction of the masseters^45^; 2) This contraction allows the mandible to become a fixed point, which gives the suprahyoid and infrahyoid muscles a role as craniocervical flexors when activated (Fig. 1). Falla and colleagues reported that, through their insertions from the mandible to the hyoid bone and from the hyoid bone to the thorax, respectively, contraction of the suprahyoid and infrahyoid muscle groups induces craniocervical flexion when the mandible is fixed.^46^

In this experimental study, our objectives were to compare in soccer players, depending on whether or not they wore this CM-MG, the head accelerations when heading, the peak force ratio of the head flexor/extensor muscles, and the acute changes in brain function associated with repetitive soccer headers, using TMS corticomotor inhibition and cognitive analyses. We hypothesised that clenching while wearing this CM-MG might be associated with decreased head accelerations when heading, and with less acute brain changes immediately after ten soccer headers. Even if we expected subtle and asymptomatic brain changes as in the Di Virgilio et al. study,^29^ we hypothesised that any decrease in the observed brain changes with CM-MG would be a favourable step before continuing studies exploring the potential protective effect of CM-MG against symptomatic brain damage in the longer term.

## Methods

### Ethics

This study was approved by the national Research Ethics Committee (n°2020-A00333-36) on March 26, 2020 and all participants included provided written informed consent prior to taking part to any study activities, according to the Declaration of Helsinki.

### Recruitments

This experimental study took place in Bordeaux (France) and the protocol was approved by the Bordeaux University Hospital (CHUBX 2019/45). Twenty-one healthy, amateur male soccer players (age 21–24 y) were recruited via meetings or contacts with local soccer clubs (Supplementary Material: Fig. 1). The selection criteria were to have been playing soccer for at least 10 years, to have learnt to head a ball in a soccer club and to head a ball regularly during soccer practice. Participants were excluded from taking part if they presented with any of the following: 1) history of Traumatic Brain Injury (TBI), whatever its severity, or any other neurological, psychiatric or progressive disease; 2) Abnormal neurological examination; 3) Use of psychoactive recreational or prescription drugs, or excessive alcohol consumption (>20 g of alcohol per day). The medical consultation was followed by a standard neurological examination to ensure that there were no abnormalities. Sex data were self-reported by study participants. Inclusions took place from June 4, 2020 to October 7, 2020, and date of the last visit was January 15, 2021. Two participants withdrew from the study for personal reasons and one participant did not sufficiently demonstrate the required technique for heading, so his participation was stopped at the first visit without post-heading assessments being conducted. The final cohort included a total of 18 participants (age 22 ± 3 y; weight 75·6 ± 9·6 kg; height 180·3 ± 5·11 cm; 14 right-handed). The median number of years of soccer playing was 17 years (16; 18), and the median number of years of education was 16 years (16; 17) (Supplementary Material: Table 1). Only male players were included because in this exploratory study we wanted a homogeneous group comparable to that of the Di Virgilio et al. study.^29^

### Study design

Following the inclusion visit with medical exam, the participants had three visits with a dentist to make the CM-MG, then they were invited to perform two experimental sessions separated by at least one week. Participants were asked to refrain from vigorous physical activity, consuming alcohol and caffeine or smoking for 24 h prior to each experimental session. For each of the two experimental sessions, neck muscle peak force measurements were recorded and baseline (pre-heading) data were acquired, including both cognitive data and electromyographic (EMG) recordings combined with TMS. Then, participants performed the heading protocol on a motion analysis platform to record kinetic data. Immediately after the heading protocol, cognitive and electrophysiological measurements were recorded again. The data used or presented in this paper have been generated according to the initial protocol available in the Supplementary material. Keeping in mind that some measurements were not generated for feasibility difficulties, and that some other assessments generated or being analysed will be discussed in future publications.

### Custom-made mouthguard (CM-MG) fabrication

**Fig. 2:**
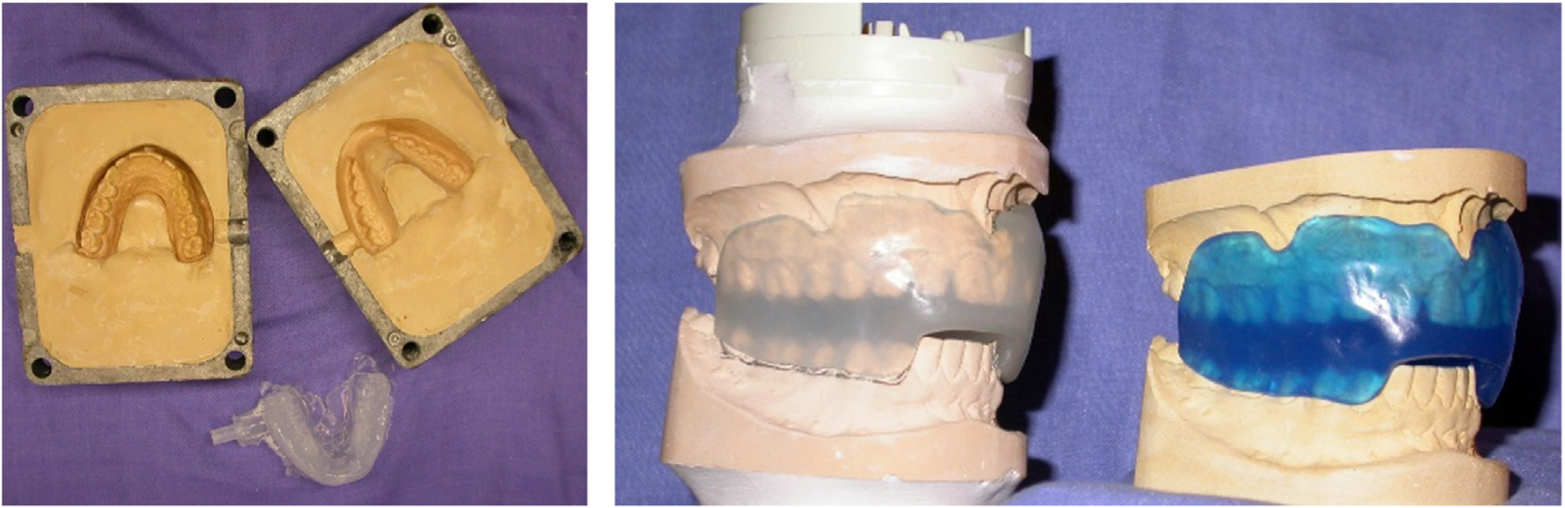
**A custom-made mouthguard (CM-MG) was made by injection technique for each participant.** Photo on the left shows preserved casts and CM-MG after the injection technique, and photo on the right shows finished CM-MGs. The particular concept of this CM-MG is based on the use of maxillary wax and the procedure for recording the mandibular position/wedging.

For each subject, after oral examination if necessary, followed by oral care, a CM-MG was made using the procedure described by Poisson and colleagues^47^: take impressions of both dental arches, make dental models from the impressions, make a registration wax to the maxillary model, record temporo-mandibulo-maxillary position by the specific wax using a relaxation method,^48^ make CM-MG by injection technique (pressure machine J100 Evolution™ (Pressing Dental, Euromax, Monaco), PVAc-PE copolymer cartridge Corflex orthodontic™ (Pressing Dental, Euromax, Monaco) injected at 160 °C for 15 min according to the manufacturer's recommendations), finally try, control, and adjust CM-MG (Fig. 2). Mechanical properties of PVA-PE polymers are relevant for the manufacture of CM-MGs, which are stiffer than elastomer and absorb a relatively large amount of energy for small deformations of the sample.^49^

### Heading protocol

**Fig. 3:**
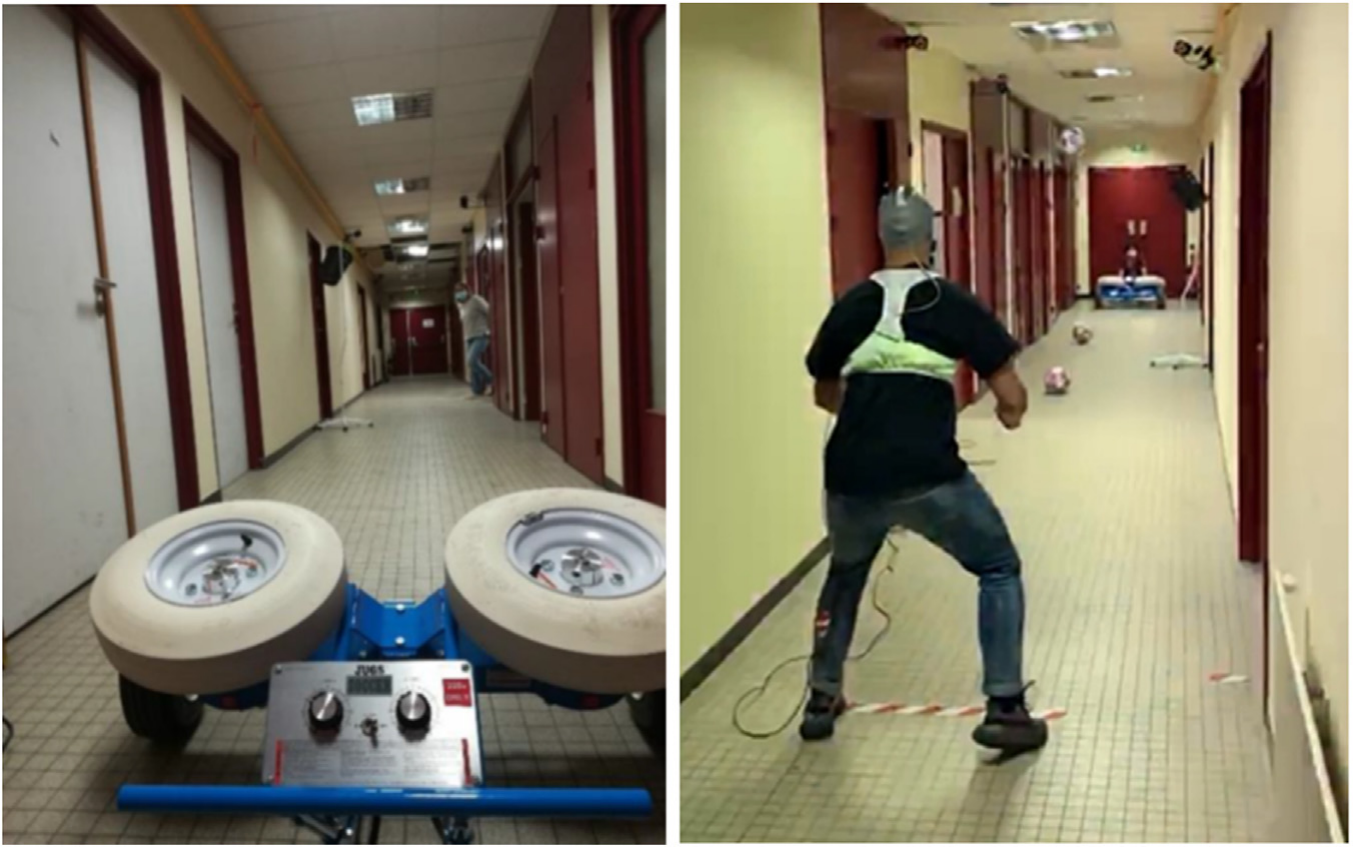
**The soccer delivery device and a soccer player during an experimental session of heading**. Participants were asked to perform ten straight headers from a standard soccer ball projected by a device (JUGS sports, Tualatin, USA) positioned 6 m away from them, redirecting the soccer ball toward a target placed 4 m in front of them. Here, the soccer player is about to kick the ball that has just been thrown.

The heading protocol consisted of heading a standard soccer ball (400 g; 70 cm circumference; 8 psi) projected at a speed of 45 ± 2·1 kph from a soccer delivery device (JUGS sports, Tualatin, USA) positioned 6 m from participants, simulating routine soccer game-play.^50^^,^^51^ Participants were instructed to perform a straight header, redirecting the soccer ball in direction to a target placed 4 m away from them, shifted 1 m to the right from the initial trajectory of the ball. Each session consisting of ten consecutive head impacts over a 20 min period. The two experimental sessions differed in oral conditions during the heading protocol: a condition without CM-MG and clenched jaws, and a condition with CM-MG and clenched jaws (Fig. 3). There was an interval of at least one week between the two experimental sessions. Half of the group completed the “without CM-MG” session first, and the other half completed the “with CM-MG” session first.

### Acquisition of kinetic data during heading protocol

A 3-Axis Digital Accelerometer (±200 g, Grove ADXL372, 1365 Hz) placed at the back of the participant's head recorded linear acceleration of the head during impact (the sensor was attached by a swimming cap at the occiput, to prevent it from moving during frontal or overhead impacts with the ball). The velocities of the ball and the head at the time of impact were calculated using kinematics extracted from five Optitrack S250 cameras (NaturalPoint, Corvallis, Oregon) fixed to the ceiling, which recorded the positions of reflecting markers that were placed on the bathing cap and on the ball at 250 Hz.

Data extraction consisted of retrieving the x, y and z coordinates of the head and ball markers. The 3D linear accelerations (expressed in gravitational unit (1 g = 9·81 m/s^2^)) were calculated using the following formula: (acceleration_x^2^+acceleration_y^2^+acceleration_z^2^):^1/2^. The detection of headers was done on 3D linear acceleration: a header is detected if the value exceeds 2∗median (Acceleration3D). The peak acceleration in the half-second following this point is then sought, making it possible to obtain the Peak acceleration of the head during impact.

### Neck muscles peak force assessment

**Fig. 4:**
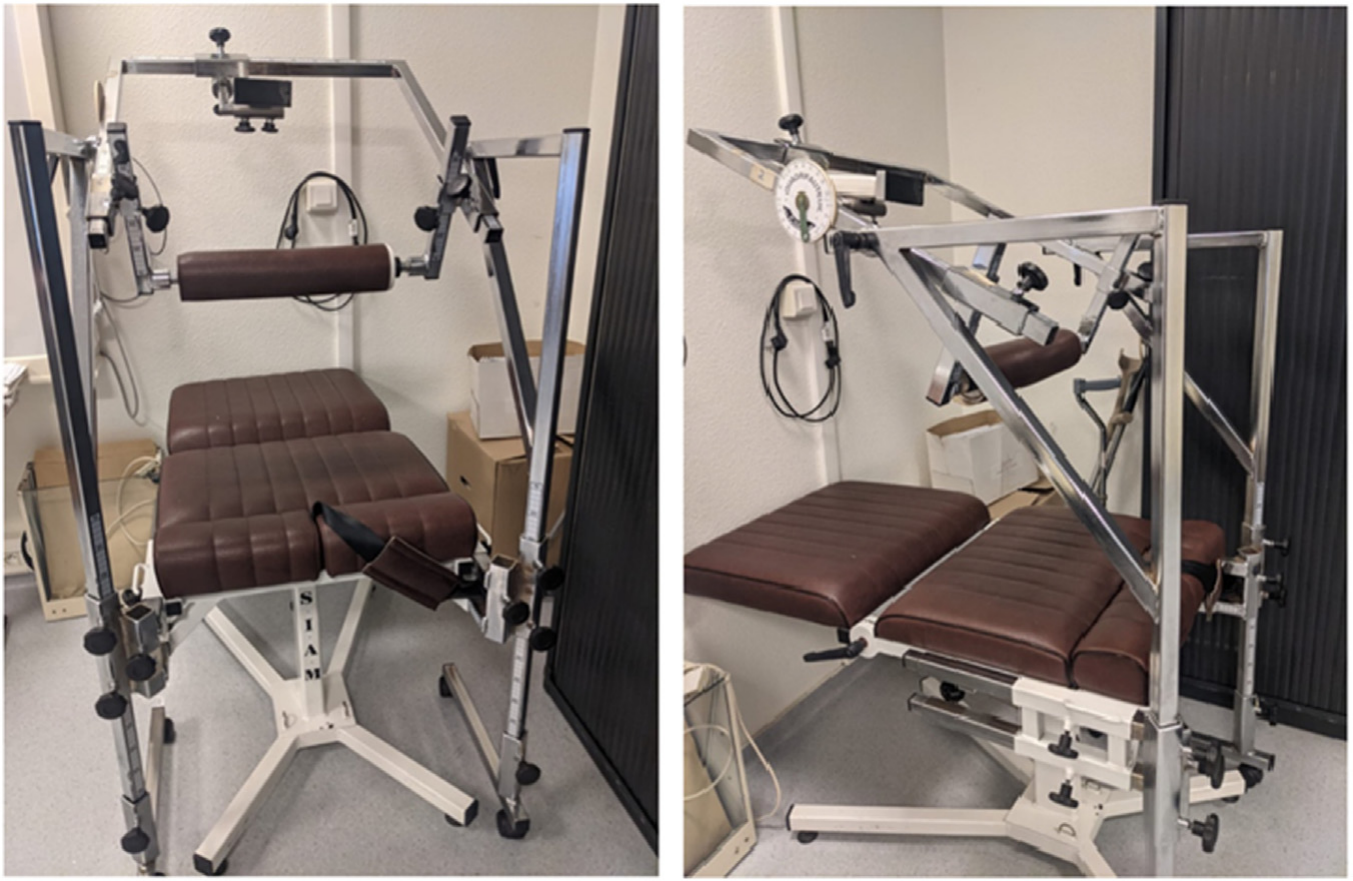
**Equipment with isometric dynamometer device, used to measure neck muscle peak force**. Participants were installed seated, with their trunk in a neutral position at 90°. The dynamometer was applied to the forehead to measure the peak force of head flexors, then it was positioned on the occiput to measure the peak force of head extensors.

Neck muscle peak force was assessed using an isometric dynamometer device, comprising different removable and adjustable parts (Fig. 4).^52^ Participants were installed seated on specific equipment, with adjustable backrest. The seat had a removable metal frame on which was integrated a dynamometer, which could be oriented forwards or backwards, with different possible angles to adapt to the size and build of the participants. For all the measurements, the participants had their trunk in a neutral position at 90°, their back and torso wedged against the back of the seat and supported forwards. The dynamometer was applied to the participants' forehead to measure the peak force of their head flexors, then it was positioned on their occiput to measure the peak force of the head extensors. For both positions, the players were asked to perform a maximum isometric contraction three times, with 30 s of rest between two trials. The maximum peak force for each of the three trials was collected. These measurements were performed under two different conditions: a condition without CM-MG and a condition with CM-MG, according to the corresponding experimental session.

### Cognitive measurements

Cognitive assessment was conducted in a quiet room in the two experimental sessions, before and immediately after each session of heading protocol, using a computerised tool on an iPad: the Cambridge Automated Neuropsychological Test Battery (CANTAB). We used the 5 subtests usually performed in research on head injury and cognitive effects in healthy volunteers,^53^^,^^54^ four of which were used in the study by Di Virgilio and colleagues in soccer players.^29^ The total duration was approximately 40 min, including assessments of sustained attention (Rapid Visual Processing (RVP)), spatial working memory (SWM), episodic memory (Paired Associates Learning (PAL)), mental flexibility (Multitasking test (MTT)), and visual planning, reasoning, impulsivity (One Touch Stockings of Cambridge (OTS)).

### Transcranial magnetic stimulation and electromyography

Motor evoked potentials (MEPs) were elicited in the flexor pollicis longus muscle of the dominant upper limb via single pulse TMS and assessed using EMG recordings. Single magnetic stimuli were applied over the contralateral primary motor cortex using a magnetic stimulator (MagPro X100unit, MagVenture®) and a 75 mm 8-shaped coil (Cool-B65 Butterfly Coil, MagVenture®). Optimal coil location for generating MEPs was determined by placing the coil over the motor cortex, laterally to the vertex; the area where the largest MEP peak-to-peak amplitudes occurred was identified and memorised using Neuronavigation with TMS Navigator Software (Localite®).

EMG activity was recorded using a PowerLab® 4/30 system (ADInstruments Pty Ltd). Data were sampled at 2 kHz, and filtered using 500 Hz low and 1·0 Hz high band filters. EMG activity was assessed using surface electrodes with an intra-electrode distance of 2 cm positioned over flexor pollicis longus; prior to electrode placement, the area of interest was shaved and abraded. The position of each electrode was marked with permanent ink to ensure consistent placement during each assessment. We chose the flexor pollicis longus because of its superficial projection on motor homunculus, requiring lower impulsions than rectus femoris (muscle used in Di Virgilio et al. study). The Active Motor Threshold (AMT) was determined for each participant by increasing stimulator by 10%, while the participant held an isometric Maximal Voluntary Contraction (MVC) until discernible MEPs were visible. Then, subsequent stimulations were delivered at 130% of AMT.

Corticomotor inhibition was measured during participant performs maximal pollicis flexor MVCs of 5 s while a single TMS stimulation was delivered over the motor cortex. This was repeated five times with a minute's rest between each contraction.

Corticomotor inhibition was quantified as the cortical silent period (cSP) duration, i.e. the period of EMG silence after a MEP, visually and manually taken from the stimulation artefact to the resumption of discernible, uninterrupted EMG activity from the muscle, on the LabChart® 7·3·7 software. All curves were analysed by the same rater. Corticomotor inhibition has a good inter-day reliability with coefficient of variation (CV) values statistically lower than those for corticospinal excitability.^55^ In the literature, to quantify the duration of the cSP on the curves, some researchers use visual analysis and others use mathematical threshold approaches compared to mean EMG amplitude. Damron and colleagues compared these two approaches and it seems that visual analysis has a lower variability than automated methods, with a between–visit coefficient of variation (CV) which was less for the visual methods and an inter-rater reliability very high. These results suggest that the manual error associated with the subjective visual analysis is relatively minimal.^56^ These measures were realised before and immediately after a series of ten consecutive head impacts with and without the CM-MG.

### Statistics

Data are expressed as mean ± standard deviation (SD) or median and interquartile range [Q1; Q3]. To compare the variables, we used either a Student's t test for paired data if the variables followed a normal distribution, or a Wilcoxon signed rank test.

For EMG data, we calculated mean cSP for each participant from the stimulation artefact to the resumption of discernible, uninterrupted EMG activity. Cognitive measures and mean cSP values acquired before and immediately after the heading protocol were compared, and we calculated the mean difference between pre an post heading for each of the two conditions, without and with CM-MG.

Each participant was considered as his own control, so we compared the following variables under the two different conditions, without CM-MG and with CM-MG: a) Mean linear acceleration of the head during the ten head-to-ball impacts (primary outcome), calculated for each participant from the Maximum Peak acceleration of each impact; b) Peak force measurements and the force index (ratio of peak force to body weight) of the head flexors and extensors, as well as the peak flexor/extensor force ratio; c) Mean difference in cognitive measures between pre an post heading; d) Mean difference in mean cSP between pre and post heading.

Confidence intervals (CI) below and above 95% were calculated from the difference in mean or median values. The significance level was set at 5%. Effect sizes (ES) for the median difference in mean cSP were calculated dividing the Z statistic by the square root of the sample size and were quantified as follows: 0·2 = small; 0·5 = medium; 0·8 = large. All analyses were performed using R software.

The number of participants was defined based on the studies closest to the proposed protocol. On the one hand, the study by Di Virgilio and colleagues (2016) which showed changes in neurophysiological functions and cognitive performance immediately after a series of intentional headers made by a group of 19 amateur soccer players aged 19 to 25.^29^ In the other hand, the studies which highlighted a decrease in linear acceleration during head impacts while playing rugby with mouthguard among 12 rugby players,^37^ and during heading in 11 soccer players when clenching while wearing a CM-MG.^38^ Using the data published in the article by Narimatsu et al., comparing head acceleration during heading in 11 soccer players between different oral conditions (mean = 28·4 g with SD = 7 for the condition “without clenching and without MG”, and mean = 21·5 g with SD = 4·6 for the condition “Clenching while wearing a MG”),^38^ we calculated that with a sample size of 18 participants, the study would have 80% power at a 5% level of significance to detect an effect size of d = 1·23 in the primary outcome. This corresponds to a large effect size according to Cohen's conventions.

### Role of funders

The study sponsors had no involvement in study design; in the collection, analysis, and interpretation of data; in the writing of the report; and in the decision to submit the paper for publication. No author has a financial relationship with the device tested here.

## Results

### Peak linear accelerations of the head during heading protocol with and without CM-MG

The average speed of the ball before head impact during the ten headers did not differ statistically significantly between the conditions without CM-MG (13·12 ± 3·04 m/s) and with CM-MG (12·71 ± 1·42) (p = 0·09; two-sided paired t-Test).

The mean peak linear acceleration of the head was statistically significantly higher without CM-MG (34·15 ± 16·97 g) than with CM-MG (26·43 ± 9·52 g) (p = 0·01; Wilcoxon signed rank test), with a median difference of 6·40 g (95% CI [−11·74; 39·09]).

### Neck muscles peak force with and without CM-MG

**Fig. 5:**
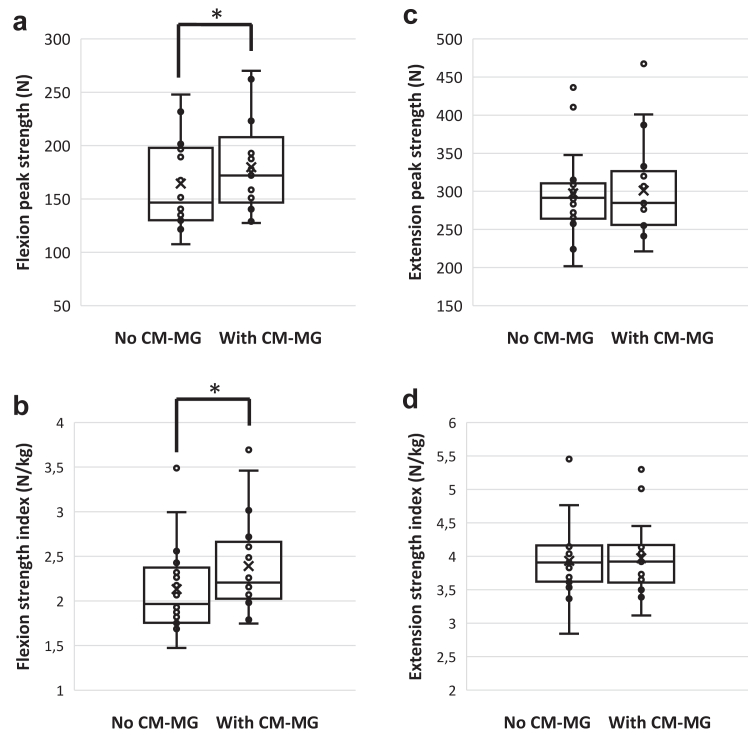
**Median difference between pre and post heading in peak force of neck muscles and force index for head flexion and head extension, without and with custom-made mouthguard (CM-MG)**. N = 18. Error bars indicate interquartile intervals, and “x” indicates the mean. A statistically significantly higher peak force generated by the head flexors was found with CM-MG (on the right) than without (on the left) (a), with a mean difference of 19·33 N (95% CI [13·39; 25·27]) (∗p < 0·001; two-sided paired T-Test), and also when related to weight (Force Index) (b), with a mean difference of 0·26 N/kg (95% CI [0·18; 0·34]) (∗p < 0·001; two-sided paired T-Test). There was no statistically significant difference in peak force generated by the head extensors (c) or in extension force index (d) between conditions with or without CM-MG.

A statistically significantly higher peak force generated by the head flexors was found with than without CM-MG, with a mean difference of 19·33 N (95% CI [13·39; 25·27]) (p < 0·001; two-sided paired T-Test), and also when related to weight (Force Index), with a mean difference of 0·26 N/kg (95% CI [0·18; 0·34]) (Fig. 5). There was no statistically significant difference in peak force generated by the head extensors between conditions with or without CM-MG. The peak flexor/extensor force ratio was significantly higher with than without CM-MG (p = 0·0026; two-sided paired T-Test), with a mean difference of 0·06 (95% CI [0·02; 0·09]).

### Effect of heading with and without CM-MG on cognitive function

Without CM-MG, a decrease in memory performance was found after the heading protocol compared to baseline, with a statistically significant increase in the mean number of errors in the spatial working memory (SWM) test (p = 0·023; Wilcoxon signed rank test) with a median difference of 0·5 (95% CI [−3·0; 31·63]) and in the Paired Associates Learning (PAL) test (p = 0·008; Wilcoxon signed rank test) with a median difference of 2 (95% CI [−2·0; 7·58]). With CM-MG, no statistically significant difference before and after the heading protocol was found in the memory performance (Supplementary material: Table 2). The difference in the mean number of errors between pre an post heading decreased statistically significantly with CM-MG than without for SWM test (p = 0·011; Wilcoxon signed rank test), with a median difference of −3·5 (95% CI [−26·63; 7·45]) and for PAL test (p = 0·018; Wilcoxon signed rank test), with a median difference of −2 (95% CI [−8·73; 1·0]) (Table 1).

### Effect of heading with and without CM-MG on corticomotor inhibition

**Fig. 6:**
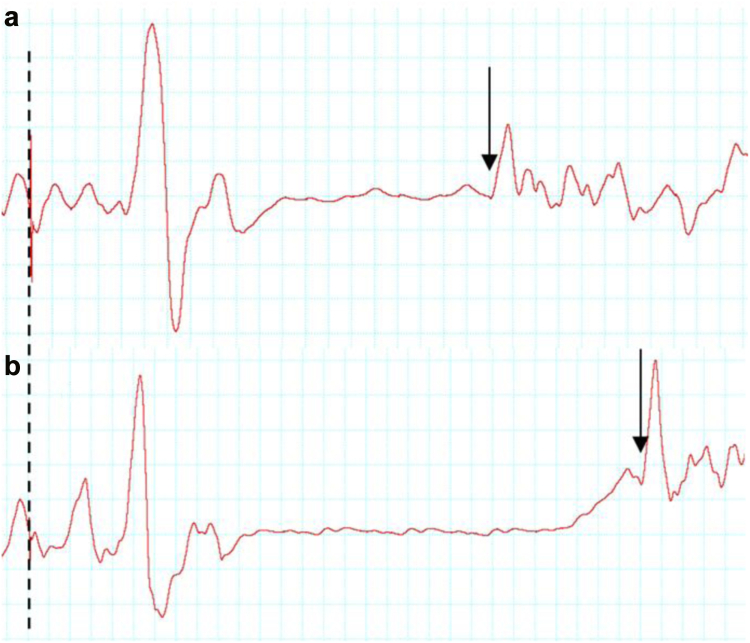
**Snapshots of the cortical silent period (cSP) of one participant measured by TMS before (a) and immediately after the heading protocol (b), illustrating increase in cSP immediately following heading**. The cSP was quantified as the period of time between the delivered TMS pulse (dashed line) and the resumption of uninterrupted EMG activity (arrows).

**Fig. 7:**
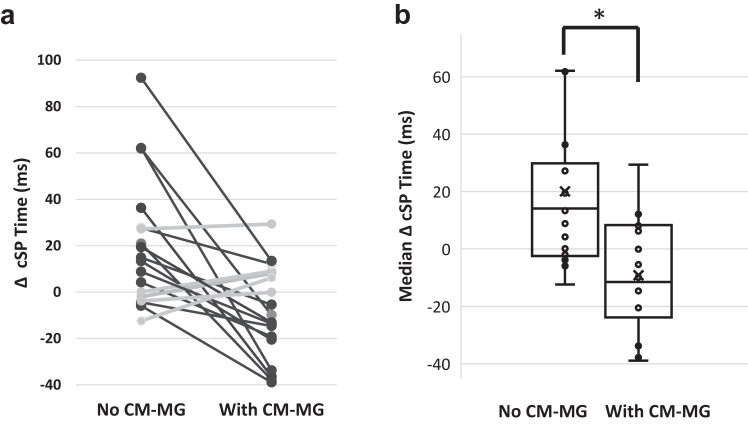
**Difference for each participant (a) and median difference (b) between pre and post heading in cortical silent period (cSP) time, without and with custom-made mouthguard (CM-MG)**. N = 18. a) The difference in cSP measure between pre and post heading decreased (in black) within 14 of the 18 participants in the condition with CM-MG (on the right) compared to with no CM-MG (on the left). b) Error bars indicate interquartile intervals, and “x” indicates the mean. The difference in median cSP time between pre and post heading was statistically significantly lower with CM-MG (on the right) than without (on the left) (∗p = 0·0016; Wilcoxon signed rank test).

Without CM-MG, immediately after the heading protocol, there was a measurable increase in cSP measure within 77·8% (14/18) of participants compared to baseline, representing an increase of 14·6% in mean cSP, statistically significant (p = 0·004; Wilcoxon signed rank test; ES 0·743; median difference of 12·02 with 95% CI [−11·48; 66·64] (Fig. 6). With CM-MG, no statistically significant change in cSP mean duration was found (p = 0·067; Wilcoxon signed rank test; ES 0·497) (Supplementary material: Table 3). The difference in mean cSP between pre and post heading decreased statistically significantly with CM-MG than without (p = 0·0016; Wilcoxon signed rank test), with a median difference of −22·87 ms (95% CI [−45·85; −4·02]) (Table 1, Fig. 7).

**Table 1:** Changes between pre and post heading (mean difference ± standard deviation) in cognitive measures and in cortical silent period (cSP) duration, in the two conditions without or with CM-MG, and 95% lower and upper confidence intervals (CIs) for the median difference between these two conditions.

Variable	Without CM-MG	With CM-MG	Δ median (95% CI)	p-value^a^
**Cognitive function**				
MTT MDL (ms)	6·8 (±24)	27·5 (±27·7)	17 [−38·2; 102·6]	0·07
PAL TEA	2·33 (±3·29)	−0·17 (±1·82)	−2 [−8·73; 1·0]	0·011
SWM TE	6·61 (±11·23)	0·44 (±8·50)	−3·5 [−26·63; 7·45]	0·018
OTS MDLFC (ms)	1387 (±3065)	−587 (±3354)	−1346 [−9287; 2706]	0·054
RVP MDL (ms)	−9·28 (±27·6)	−1·39 (±43·5)	12·25 [−49·9; 51·7]	0·31
**Corticomotor inhibition**				
cSP (ms)	20·03 (±28·01)	−9·17 (±19·92)	−22·87 [−45·85; −4·02]	0·0016

N = 18.

Multitasking Task: Median reaction Latency (MTT LMD); Paired Associates Learning: Total Errors Adjusted (PAL TEA); Spatial Working Memory: Total Errors (SWM TE); One Touch Stockings of Cambridge: Median Latency to First Choice (OTS MDLFC); Rapid Visual Processing: Median response Latency (RVP MDL). Custom-Made-Mouthguard (CM-MG).

a Wilcoxon signed rank test.

## Discussion

This experimental study found in this cohort a significant decrease in peak linear head acceleration of soccer players during the heading protocol when wearing a CM-MG compared to the standard condition without CM-MG, and an increase in peak force of the head flexor muscles when wearing the CM-MG.

Furthermore, our results showed in this cohort that the changes in cSP and memory performance immediately found after performing ten consecutive soccer headers compared to baseline assessments decreased statistically significantly when wearing the CM-MG than when not wearing it.

While the mean peak linear acceleration of the head found with or without CM-MG remained below the concussion risk thresholds (80 g–89 g depending on the studies),^2^^,^^57^ we don't know if the difference of 6·4 g found between the two conditions could be clinically significant when multiplied by the hundreds or thousands of headers performed over several years of soccer practice. Furthermore, we used an 8 psi ball in this study, meaning head impact forces were expected to be considerably reduced compared to the more usual 16 psi that FIFA has mandated.^58^ Thus, our findings seem to support those of a previous study that also found a statistically significant decrease in linear accelerations during headers in 11 soccer players when clenching while wearing a CM-MG, compared to clenching without this mouthguard and even more compared to no clenching and no mouthguard.^38^ In this study, after the players were instructed to clench their masseter muscles, a decrease in head acceleration was observed. The effect was strengthened when players clenched with the CM-MG, resulting in significant increases in masseter and sternocleidomastoid muscle activity. Evidence of a significant increase in peak head flexor force when wearing the CM-MG, and therefore a more balanced force ratio between the head flexors and extensors with this device, potentially further reinforced by jaw clenching during heading, could partly explain the changes in acceleration of the head. However, further comparative studies will be necessary with a standard mouthguard to verify whether the effect is the same.

In our study, when the soccer players didn't wear the CM-MG during heading, the findings on corticomotor inhibition and cognitive data were in agreement with those of the experimental study conducted by Di Virgilio and colleagues, which found a transient increase of cSP duration and a transient decrease in memory performance in soccer players immediately after twenty consecutive headers.^29^ This suggest that these changes would also be present after performing only ten consecutive headers, which is closer to the average number of headers performed per player in a match. We did not repeat the measurements later after the heading protocol, as the changes did not persist beyond 24 h in the Di Virgilio and colleagues' study.^29^

To investigate corticomotor inhibition in their study,^29^ authors had chosen a target on the lower limb while ours was on the upper limb. Knowing that the motor cortex corresponding to the upper limb is more superficial than the one corresponding to the lower limb, this may explain why the cSP differential between pre-et post-heading was even greater in our study without CM-MG. The electromyographic cSP measured is in part mediated by GABAb receptors, GABA being the most potent inhibitor of the motor system. GABAb receptors appear to play an important role in long term potentiation (LTP) mechanisms of brain plasticity and learning. Furthermore it has been demonstrated that baclofen, a GABAb receptor agonist, abolishes LTP-plasticity.^59^ Some authors have suggested that increase in corticomotor inhibition could be related to excessive neurotransmission of GABAb in the motor cortex of soccer players exposed to repetitive head impacts and conduce to a dysregulation of LTP mechanism of brain plasticity.^60^ It has been suggested that LTP alteration in brain plasticity can be partly related with motor learning impairments in a group of Canadian University football players with a history of repeated concussions.^61^ GABAb probably plays its inhibitor role via blockade of NMDA receptors-mediated glutamatergic expression. The inhibitory action of GABA might play a neuroprotective role against the excitotoxicity of glutamate (molecule released in excess during concussion), therefore against cell death. The neuroprotective role of GABAb in the development of a metabolic state of resistance to cerebral ischaemia in rats was suggested by neuronal analysis of organotypic cultures of hippocampal slices.^62^ Given that head impacts can cause anoxic ischaemic white matter injury, it is questionable whether a similar phenomenon could occur after concussive and sub-concussive brain injuries. Studies have shown that exercise too soon after concussion can lead to adverse consequences due to increased inflammatory action, increased excitotoxic effects of glutamate, and decreased neuroprotective mechanisms.^63^ Further studies are needed to better understand the relationship between GABAergic neurotransmission and head impacts symptoms.

Furthermore, cognitive changes post heading without CM-MG were found in our study, with a decrease in spatial working memory and episodic memory performance in the two same CANTAB® subtests as in Di Virgilio and colleagues’ study. While a normalisation of performance in subsequent follow-up assessments at 24 h, 48 h and 14 days in their study, poorer cognitive performance had ever been reported in retired soccer players, particularly in memory, executive functions and attention.^64^ A relationship has also been found between memory function and the history of the cumulative number of headers in former amateur soccer players, with an estimated threshold of 1800 headers per year to cause memory problems.^22^ However, these observations remain controversial and the recent meta-analysis by Kontos does not establish a link between heading and persistent cognitive impairment, mainly due to the existence of numerous biases and different methodologies that do not allow for comparison between studies.^65^

Otherwise, in both Di Virgilio and colleagues’ study and our study, mental flexibility, sustained attention and processing speed appeared to be preserved from any acute changes associated with heading, compared to baseline assessments. The test added in our study to assess other executive functions such as visual planning, reasoning and impulsivity also found no significant difference before and after heading. In addition, in our study, the differences in performance on the visuo-spatial working memory and visual episodic memory tasks before and after the series of ten headers were reduced in the CM-MG condition, which suggest that CM-MG might prevent the onset of transient post-heading memory performance alteration.

Nevertheless, this study had some limitations. This experimental heading model does not reflect all the heading situations that can be observed in the field. For our study, the heading protocol had to be reproducible and comparable between each trial and each participant. However, head accelerations vary depending on the heading technique as well as the approach, action scenario and player intention.^66^ We therefore deliberately defined identical extrinsic factors (angle and speed of projection, ball pattern and inflation), with a single heading scenario, repeated and always associated with the same instructions, in order to ensure reproducibility of measurements and to be able to compare conditions with and without mouthguard. Furthermore, only linear acceleration was recorded in our protocol, and tracking the motion of the head using sensors affixed using caps is prone to errors.^67^ Therefore, future research will use an instrumented CM-MG to study skull acceleration, as mouthguards have shown tighter skull coupling, and will include analysis of angular accelerations.^67^ Another limitation was the small number of participants which is explained by the exploratory design of this study. In addition, we chose a very targeted population in order to have a homogeneous group, including young men with an average age of 23 years, who played amateur soccer for a median period of 17 years. The results cannot therefore be extrapolated to other player profiles, in particular different soccer experience levels, females (a growing population in this sport), and other age groups, especially since the neck muscle force ratios in these populations are not comparable.

Finally, these results demonstrate that the CM-MG was associated with increased head flexor muscle peak force in this cohort, improving balance between head flexors and extensors, and support our hypotheses that wearing a CM-MG would reduce soccer players' peak head acceleration during headers, and could be protective for brain function. Reducing head acceleration during sports-related head impacts might be crucial for preventing degenerative brain injuries. Future studies exploring the brain effects of soccer heading when wearing and not wearing CM-MGs in all playing conditions on the field are needed.

## Contributors

HC, BG, PP and PD were responsible for the conception and design of the study. CP, PL, ED and EG was responsible for data collection and treatment. PP made the custom-made mouthguards. PL, CP and HC conducted the statistical analyses. CP, PL and HC wrote the paper. The draft of the paper was critically revised by PP, EG, ED, BG, HC and PD. All authors read and approved the final version of the manuscript. HC, CP and PL have accessed and verified the underlying data.

## Data sharing statement

The data that support the findings of this study are available from the corresponding author (HC), upon reasonable request: helene.cassoudesalle@chu-bordeaux.fr.

## Declaration of interests

None declared.
