# Supplementary material for: A custom-made mouthguard reduces head acceleration during soccer heading and prevents acute electrophysiological and cognitive changes in amateur male players
Source: eBioMedicine. 2025 Apr 2;115:105674. doi: 10.1016/j.ebiom.2025.105674 (PMC11999492; doi:10.1016/j.ebiom.2025.105674)
Supplement: Supplementary Figure and Tables [file mmc1.pdf]

Supplementary Material

Figure 1: Flow diagram of participants.

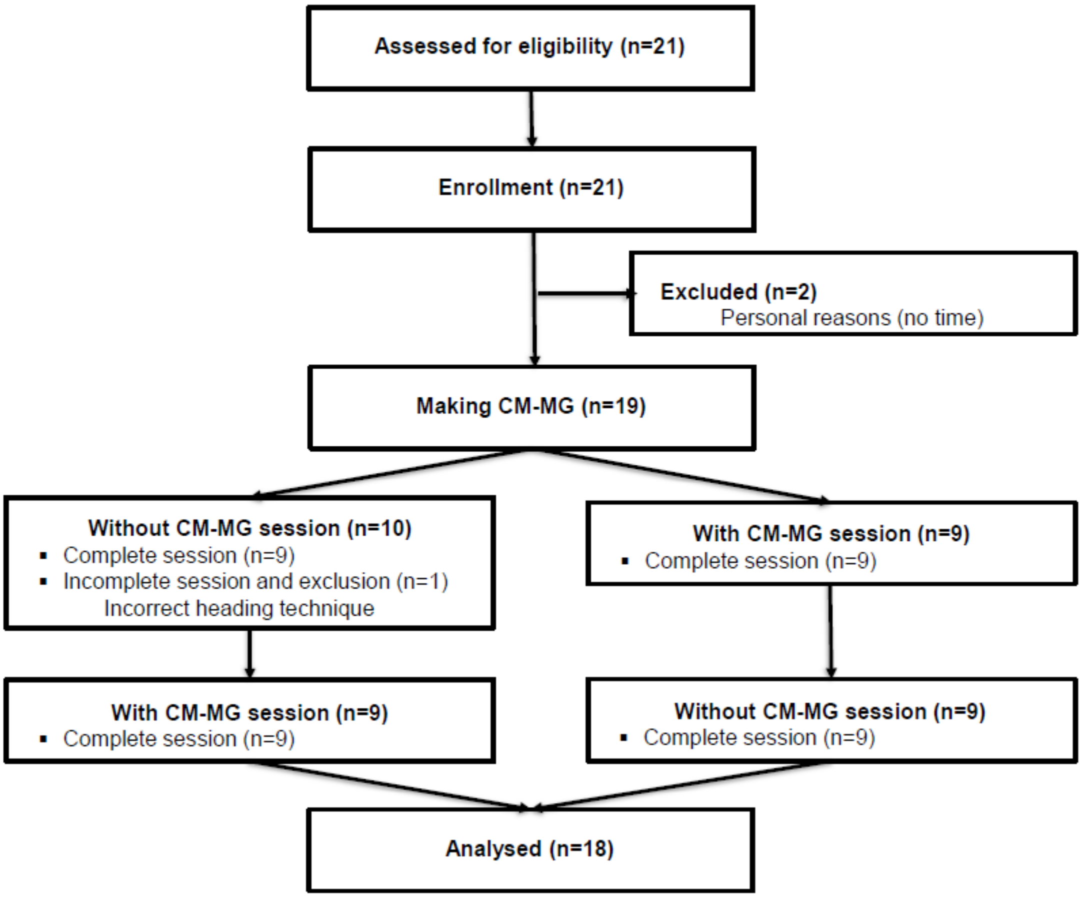

Table 1: Baseline characteristics of participants included in data analyses (n=18).

Soccer Players	Age (years)	Years of education	Years of soccer playing	Hand laterality	Height (cm)	Weight (kg)
Player 1	21	16	14	Left	179	71
Player 2	23	16	18	Right	180	74
Player 3	23	16	17	Right	178	73
Player 4	23	17	17	Left	176	63
Player 5	23	16	18	Left	186	78
Player 6	23	15	17	Right	187	80
Player 7	22	16	17	Right	183	73
Player 8	23	15	18	Right	186	89
Player 9	23	16	18	Right	170	62
Player 10	23	16	18	Right	185	76
Player 11	23	17	15	Right	180	69
Player 12	23	17	12	Left	175	72

Player 13	23	17	18	Right	178	70
Player 14	23	17	18	Right	178	105
Player 15	23	17	17	Right	180	77
Player 16	22	17	17	Right	190	73
Player 17	24	17	16	Right	181	78
Player 18	21	15	18	Right	174	77

Table 2: Mean value ± standard deviation for each measure of the CANTAB® subtests. N=18.

	Without CM-MG			With CM-MG		
	Pre-heading	Post-heading	p-value	Pre-heading	Post-heading	p-value*
MTT MDL (ms)	449·8 ±71·2	443·0 ±77·5	0·248	433·4 ±77·8	405·8 ±61·5	0·00059
PAL TEA	1·83 ±1·65	4·17 ±2·66	0·008	1·94 ±1·83	1·78 ±1·56	0·7
SWM TE	6·78 ±8·29	13·39 ±14·21	0·023	6·33 ± 7·33	6·78 ±9·25	0·83
OTS MDLFC (ms)	7005 ±1305	8392 ±2448	0·0562	7394 ±2927	6807 ±2092	0·46
RVP MDL (ms)	408·8 ±28·9	399·5 ±34·9	0·17	398·4 ±43·52	397·0 ±35·5	0·89

Multitasking Task: Median reaction Latency (MTT LMD); Paired Associate Learning: Total Errors Adjusted (PAL TEA); Spatial Working Memory: Total Errors (SWM TE); One Touch Stockings of Cambridge: Median Latency to First Choice (OTS MDLFC); Rapid Visual Processing: Median response Latency (RVP MDL). Custom-Made-Mouthguard (CM-MG).
*Wilcoxon signed rank test

Table 3: Mean ± standard deviation values for cortical silent period (cSP) duration in ms, and 95% lower and upper confidence intervals (CIs) for the mean difference between pre and post heading. N=18.

Condition	Baseline	Post-heading	Δ mean pre vs post (95% CI)	p-value*
Without CM-MG	115·6 ± 26·9	132·4 ± 35·6	16·84 [28·5 to 5·2]	0·004
With CM-MG	118·8 ± 28·4	102·4 ± 25·4	-9·436 [0·8 to -19·7]	0·067

*Wilcoxon signed rank test
